# TEMPO-Oxidized Nanocellulose In Situ-Immobilized AgNPs-Modified Chitin-Based Composite Sponge for Synergistic Antibacterial Fruit Preservation

**DOI:** 10.3390/polym18030327

**Published:** 2026-01-26

**Authors:** Zijun Zhang, Qi Zhang, Qimeng Jiang, Hao Ma

**Affiliations:** State Key Laboratory of Green Papermaking and Resource Recycling, Qilu University of Technology, Jinan 250353, China

**Keywords:** nanocellulose, silver nanoparticles, chitin, composite sponge, synergistic antibacterial

## Abstract

Sponge-based preservative packaging is an emerging approach to mitigate mechanical damage to fruits and vegetables during transportation, storage, and retail. However, conventional polyurethane sponges generally lack durable antibacterial activity and are neither biodegradable nor readily recyclable. Herein, to address these limitations, silver nanoparticles immobilized on TEMPO-oxidized cellulose nanofibers (TCNF@AgNPs) were incorporated into a quaternized chitin matrix to construct a synergistic antibacterial composite sponge (QCH/TCNF@AgNPs) for fruit preservation. The composite sponge exhibited strong antibacterial efficacy against *Escherichia coli* and *Staphylococcus aureus*, together with a low cumulative release of silver species of 2.49% after 336 h. In addition, the sponge showed >50% mass loss after 36 days in lysozyme solution, indicating good enzymatic degradability. Cytocompatibility assays further confirmed favorable biocompatibility and biosafety. Notably, the composite sponge provided satisfactory preservation performance for fresh strawberries. Overall, this work demonstrates the potential of QCH/TCNF@AgNPs as a biodegradable antibacterial packaging sponge for fruit preservation.

## 1. Introduction

With increasing expectations for the quality and shelf life of fresh fruits and vegetables, traditional packaging materials are often insufficient to meet modern demands [1,2,3]. Preservative films remain one of the most widely used packaging approaches [4]; however, for delicate fruits with thin skins and low resistance to compression (e.g., strawberries), films provide limited cushioning and can be associated with substantial losses during transportation and storage, particularly under unfavorable handling conditions. Moreover, exudates from spoiled fruits create a moist microenvironment that promotes microbial growth, which further accelerates deterioration [5,6]. Therefore, there is a strong need for packaging materials that simultaneously provide mechanical buffering and active antibacterial protection, while remaining environmentally benign. Recently, biodegradable composite packaging sponges have attracted increasing interest because they can offer lightweight cushioning, tunable porosity, and low cytotoxicity, and can be engineered for diverse food-packaging applications [7,8,9,10]. Although polyurethane sponges exhibit excellent mechanical properties, they are difficult to degrade and raise environmental concerns [11,12,13]. In addition, most conventional cushioning sponges lack intrinsic antibacterial activity against fungi and other microorganisms, limiting their suitability for fresh-produce packaging. Accordingly, developing biodegradable and antibacterial composite sponges with appropriate physical properties is important for improving fruit and vegetable preservation.

Chitin is a kind of natural biodegradable polymer that is widely found in the shells of shrimp, crabs, and insects [14,15,16,17]. After quaternary ammonium modification, the quaternary ammonium salt of chitin (QCH) can exhibit limited antibacterial activity [18,19,20,21,22,23,24]. Furthermore, QCH can dissolve in different aqueous alkali solutions to form a sponge [25,26]. However, QCH sponges also have the disadvantages of hardness, low porosity, nonelasticity, and limited antibacterial activity [27,28]. Inorganic metal nanoparticles (e.g., silver, gold, and copper) have been widely investigated as physical antibacterial agents to compensate for the limited antimicrobial activity of QCH sponges [29,30,31,32,33,34,35,36]. Among them, silver nanoparticles (AgNPs) have attracted particular attention because of their broad-spectrum antibacterial efficacy and good thermal stability [37,38]. However, conventional AgNP preparation and incorporation often suffer from rapid release, nanoparticle aggregation, and the use of toxic reducing agents [39,40]. To address these limitations, TEMPO-oxidized cellulose nanofibers (TCNF) were employed as a green scaffold to in situ generate and immobilize AgNPs without additional chemical reductants or stabilizers. The flexible TCNF chains can further interact with the more rigid QCH framework, enabling the formation of a soft and elastic composite sponge with synergistic antibacterial and preservation performance.

**Figure 1:**
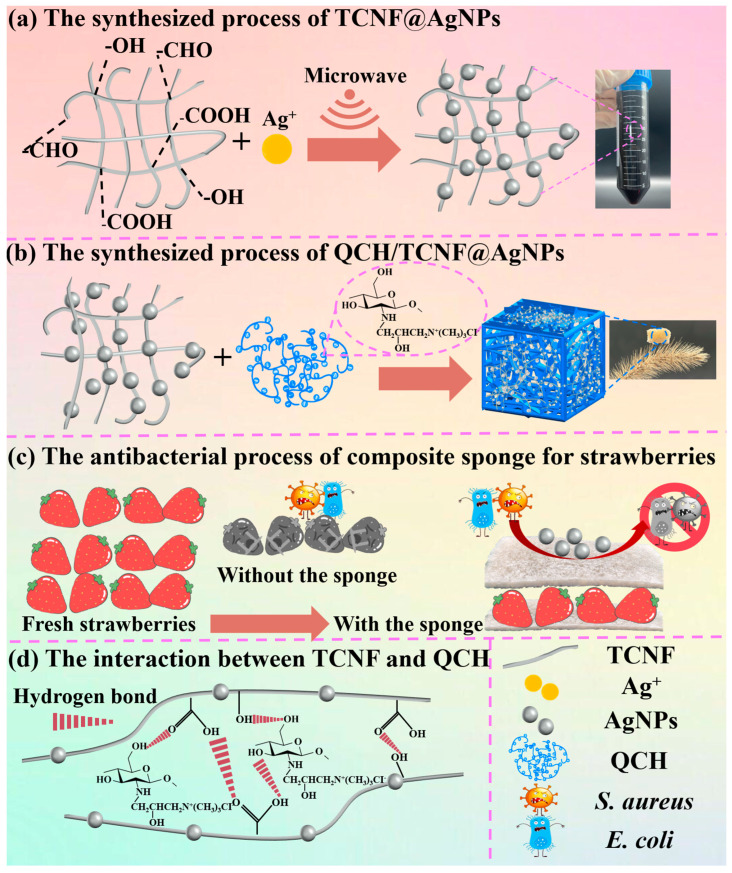
The synthesis of TCNF@AgNPs (**a**); the synthesis of QCH/TCNF@AgNPs (**b**); the antibacterial preservation process of the composite sponge for strawberries (**c**); and the interaction between TCNF fibers and QCH fibers (**d**). The structural formulae are schematics and simplified for clarity. The starting aminopolysaccharide is chitin-rich with partial deacetylation (degree of deacetylation ≤ 65%).

The key innovation of this work lies in integrating a green, reducer-free approach to generate and immobilize AgNPs on TEMPO-oxidized cellulose nanofibers (TCNF@AgNPs) with a quaternized chitin sponge, thereby producing a soft, elastic, and biodegradable antibacterial packaging sponge. Compared with previously reported AgNPs-loaded sponges that often require chemical reducing agents, suffer from nanoparticle aggregation, or exhibit burst release, our design leverages nanocellulose-assisted immobilization to promote homogeneous nanoparticle distribution and enables controlled Ag release. Meanwhile, the quaternary ammonium functionality provides complementary contact-active antibacterial action, yielding a synergistic effect while retaining cytocompatibility and environmental degradability. Specifically, as illustrated in Figure 1, schematic diagrams depict the synthesis of TCNF@AgNPs (Figure 1a) and QCH/TCNF@AgNPs (Figure 1b); Figure 1c shows the schematic of the antibacterial preservation process for the strawberry composite sponge, and Figure 1d illustrates the interaction between TCNF and QCH. This integrated strategy, therefore, offers practical advantages for fruit preservation by simultaneously providing mechanical cushioning, sustained antibacterial performance, and packaging-relevant safety.

## 2. Materials and Methods

### 2.1. Materials

TEMPO-oxidized cellulose nanofibers (TCNF; carboxyl group content: 1.20 mmol/g) were purchased from Mujingling Biotechnology Co., Ltd. (Tianjin, China). Chitin (food grade; degree of deacetylation ≤ 65%; 80 mesh) was purchased from Fengtai Biotechnology Co., Ltd. (Jinan, China). This material can be considered a chitin-rich aminopolysaccharide with partial deacetylation (i.e., containing both acetylated and deacetylated units). Sodium hydroxide (NaOH), urea, epichlorohydrin, silver nitrate (AgNO_3_), salicylic acid, ethanol, phosphate-buffered saline (PBS, pH 7.4), lysozyme, beef extract peptone agar (for bacterial culture), and glutaraldehyde (for SEM fixation) were purchased from Aladdin Biochemical Technology Co., Ltd. (Shanghai, China) and used as received for the corresponding experiments described below. *Escherichia coli* (BNCC133264) and *Staphylococcus aureus* (BNCC186335) were purchased from BeNa Culture Collection (Langfang, China). Polyurethane (PU) packaging/cushioning material was purchased from Foshan Haonengmai New Materials Technology Co., Ltd. (Guangzhou, China). Strawberries were purchased from a greenhouse fruit farm in Jinan (Changqing, China). Deionized water was used throughout.

### 2.2. Synthesis of TEMPO-Oxidized Cellulose Nanofibers-Immobilized Silver Nanoparticles

TCNF@AgNPs was prepared in situ by a microwave-assisted redox method under different conditions, as verified by our previous research [41]. In our previous study, the microwave-assisted synthesis conditions for TCNF@AgNPs were systematically optimized by varying the reaction temperature (40–100 °C), time (10–60 min), and the TCNF/silver-precursor ratio (50 mL TCNF dispersion with 0.025–0.30 g silver precursor). The present work adopts the optimized conditions (0.2 g TCNF and 0.2 g AgNO_3_ in 50 mL dispersion, 90 °C for 30 min, followed by one week of dialysis) to obtain a reproducible dispersion of TCNF-immobilized AgNPs. The TCNF (0.2 g) was dispersed into 50 mL aqueous solution using a high-pressure jet homogenizer (IKA, Staufen im Breisgau, Germany), followed by the addition of 0.2 g silver nitrate. Then, the mixture was transferred into a three-neck flask and kept for 30 min at 90 °C. Note that the above masses refer to precursor feeding amounts during in situ immobilization. After one week of dialysis to remove unreacted/loosely bound silver species, the actual silver content of the final QCH/TCNF@AgNPs sponge, determined by ICP-OES, was only 0.18 wt% (based on total dry sponge mass). After completion of the reaction, the suspension was purified by dialysis using a regenerated-cellulose dialysis membrane (MWCO: 200 Da) against deionized water for 7 days at 25 °C, with the external water refreshed four times per day to remove unreacted silver species and other low-molecular-weight impurities. The dialysis was conducted under light-protected conditions, and the purified TCNF@AgNPs dispersion was collected and stored in a brown bottle for subsequent use.

### 2.3. Fabrication of a QCH/TCNF@AgNPs Composite Sponge

A food-grade chitin material with partial deacetylation was selected because it offers a chitin-rich backbone that forms a robust porous sponge after processing, while still providing sufficient amino groups for quaternization; highly deacetylated chitosan will be explored in future work as a tunable alternative substrate. First, the quaternary ammonium salt of chitin was obtained with the raw material mass ratio of 1:0.5 (Chitin: salicylic acid) at a reaction temperature of 70 °C, a reaction time of 90 min, and a pH value of 8. Then, 13 g of quaternary ammonium-modified chitin powder was slowly dispersed into 100 mL NaOH/urea solution (mass ratios: 7%/12%) using a mechanical mixer at 500 rpm for 20 min. Next, the ultrasonic cleaning machine (Scientz-750 F, Ningbo Scientz Biotechnology Co, Ltd., Ningbo, China) was used to disperse the above solution for 20 min before the mixture was refrigerated at −13 °C for 24 h. Next, the frozen quaternary ammonium salt of chitin solution was thawed under stirring at room temperature, and the freeze–thaw process was repeated 4 times. Next, the quaternary ammonium salt of chitin solution (80 mL), TCNF@AgNPs solution (20 mL), and epichlorohydrin (10 mL) were mixed and continuously stirred at 0 °C for 5 h. The QCH/TCNF@AgNPs volume ratio and crosslinker dosage were selected to balance gel integrity and pore-structure formation with packaging-relevant antibacterial durability, which is consistent with the controlled Ag release observed in the subsequent release test. After that, the mixture was transferred into Teflon molds and refrigerated at 3–5 °C for 24 h to form composite hydrogels. Finally, the QCH/TCNF@AgNPs composite sponge was obtained after dialysis for 7 days to remove unreacted epichlorohydrin and freeze-dried (−65 °C, 120 h). For comparison, the pure quaternary ammonium-modified chitin sponge (QCH) and the quaternary ammonium-modified chitin/TCNF composite sponge (QCH/TCNF) were prepared using the same protocol as QCH/TCNF@AgNPs, including the freeze–thaw dissolution process, epichlorohydrin crosslinking, gelation at 3–5 °C, dialysis (for 7 days) to remove unreacted epichlorohydrin, and freeze-drying. Specifically, QCH was prepared by mixing the quaternized chitin solution (80 mL) with deionized water (20 mL) instead of the TCNF@AgNPs dispersion prior to adding epichlorohydrin. QCH/TCNF was prepared by replacing the TCNF@AgNPs dispersion with a TCNF aqueous dispersion (20 mL) of the same solid concentration, while keeping all other steps unchanged.

### 2.4. Characterization of QCH/TCNF@AgNPs

The surface and cross-section morphology of QCH/TCNF@AgNPs was characterized using scanning electron microscopy (SEM, Hitachi TM4000Plus, Tokyo, Japan). Energy-dispersive X-ray spectroscopy mapping images were also obtained by SEM. An X-ray polycrystalline diffractometer (Rigaku, Tokyo, Japan) was used to obtain the X-ray diffraction (XRD) pattern with a 2θ range (5° to 90°). X-ray photoelectron spectroscopy (XPS, Kratso, Manchester, UK) was performed using Mg Kα radiation (hν = 1253.6 eV) with a step size of 0.1 eV. An inductively coupled plasma emission spectrometer (ICP-OES, Agilent 5110, Santa Clara, CA, USA) was used to obtain the Ag content. X-ray computed tomography (CT) (SkyScan2211, Bruker, Billerica, MA, USA) was performed using an X-ray CT system to reconstruct the 3D porous architecture of the sponge. Collectively, SEM/EDS, XRD, XPS, ICP-OES, and X-ray CT were used to confirm the successful incorporation of AgNPs and their homogeneous distribution/attachment within the composite sponge. Unless otherwise specified, all specimens were freeze-dried (and dried to constant mass when required) prior to weighing to ensure an accurate dry-mass basis for porosity, swelling, and solubility calculations.

The porosity of the composite sponge was obtained by the ethanol replacement method. The test method was as follows: 50 mL of ethanol was poured into a 100 mL measuring cylinder, and three different types of sponge were completely immersed in it. The liquid volume of the measuring cylinder was marked after absorbing ethanol as V_1_. The volume of remaining ethanol was recorded as V_2_ after the sponge was removed. The porosity of composite sponges was calculated using Equation (1):

(1) $$\mathrm{Porosity}\;(\%)\;=\;\frac{50-V_{2}}{V_{1}-V_{2}}\times 100$$

The three different sponges were weighed separately as M. The volume of the sponge was obtained using the formula for the volume of a cylinder as V. The density of the composite sponge was calculated using Equation (2):

(2) $$\mathrm{Density}\;(g/{\mathrm{cm}}^{3})\;=\;\frac{M}{V}$$

The swelling was determined by a modified method as follows. The composite sponge was dried at 40 °C for 12 h in a blast air oven, and the initial weight of the sponge was recorded as M_0_. After that, the sponge was immersed in 50 mL of PBS solution for 2 h. After removing excess water with filter paper, the weight of the sponge was recorded as M_1_. The swelling of the composite sponge was calculated using Equation (3):

(3) $$\mathrm{Swelling}\;(\%)\;=\;\frac{M_{1}-M_{0}}{M_{0}}\times 100$$

The moisture absorption rate of the sponge was obtained by the following method: The sponge was dried in a drying oven at 60 °C for 24 h and weighed, with the mass recorded as M_0_. The weight was recorded as M_1_ after storage in a constant-temperature and humidity box (25 °C, 75% ± 5%RH) for 24 h. The moisture absorption rate of the composite sponge was calculated using Equation (4):

(4) $$\mathrm{Moisture}\;\mathrm{absorption}\;(\%)=\frac{M_{1}-M_{0}}{M_{0}}\times 100$$

Water solubility. Sponge specimens were first dried in a forced-air drying oven at 38 °C for 12 h and then cut into squares with a side length of 20 mm. The initial dry mass was recorded as M_0_ using an analytical balance. Each specimen was immersed in 50 mL phosphate-buffered saline (PBS, pH 7.4) for 24 h. After reaching saturation, the specimen was removed and gently blotted with filter paper to remove surface liquid, and then dried again at 38 °C for 12 h to obtain the residual dry mass (M_1_), and water solubility was calculated using Equation (5)

(5) $$\mathrm{Water}\;\mathrm{solubility}\;(\%)=\frac{M_{0}-M_{1}}{M_{0}}\times 100$$

Fourier transform infrared (FT-IR) spectra of the sponge were collected using an infrared spectrometer (Bruker, Billerica, MA, USA). The elastic properties and firmness of the composite sponge were measured using a texture analyzer (Stable Micro Systems, Godalming, UK). The firmness was tested three times with a P2 probe (with a diameter of 1 mm) at a measured speed of 30 mm/min.

Thermogravimetric analysis (TGA Discovery, SDT650, New Castle, DE, USA) was used to analyze the thermal stability assessment of QCH/TCNF@AgNPs under the temperature range of 30 °C to 800 °C with a heating rate of 20 °C/min. Differential scanning calorimetry (DSC) was performed to further assess the thermal stability of the composite sponges. Measurements were carried out under a nitrogen atmosphere over a temperature range of 0–300 °C at a heating rate of 10 °C min^−1^.

### 2.5. Biodegradation Evaluation

Biodegradability was evaluated by (i) a natural-soil burial test and (ii) a lysozyme-assisted enzymatic degradation test. For the soil burial test, pre-weighed, freeze-dried sponge specimens (initial dry mass, M_0_; size: 20 mm × 20 mm × 5 mm) were buried in natural soil (sieved to remove stones and debris) in covered containers and incubated at 25 °C. The soil moisture was maintained at 50–60% of water-holding capacity by periodic addition of deionized water. At predetermined intervals (7, 21, 56, 180 days), specimens were retrieved, gently rinsed with deionized water to remove adhering soil, and dried to constant mass at 40 °C before weighing (Mₜ). For the lysozyme degradation test, sponge specimens (M_0_) were immersed in lysozyme solution (1 mg/mL lysozyme in PBS, pH 7.4) and incubated at 37 °C under static conditions. The solution was refreshed every 3 days to maintain enzyme activity. At predetermined intervals (6, 12, 18, 24, 30, 36 days), specimens were removed, rinsed, dried to constant mass, and weighed as Mₜ. The degradation ratio in both media was calculated using Equation (6), where M_0_ is the initial dry mass, and M_t_ is the residual dry mass after degradation for time t:

(6) $$\mathrm{Degradation}\;\left(\%\right)=\frac{M_{0}-M_{t}}{M_{0}}\times 100$$

### 2.6. Antibacterial Analysis

Gram-negative Escherichia coli (*E. coli*) and Gram-positive Staphylococcus aureus (*S. aureus*) were used to evaluate the antibacterial properties of the composite sponges by inhibition-zone assays, OD_600_ growth measurements, and bacterial morphology observations. These two strains were chosen as representative Gram-negative (*E. coli*, BNCC133264) and Gram-positive (*S. aureus*, BNCC186335) bacteria commonly used as reference microorganisms for assessing antibacterial food-packaging materials; their distinct cell-envelope architectures enable a meaningful comparison of antibacterial efficacy.

A bacterial suspension (~10^7^ CFU mL^−1^) was prepared using standard cultivation procedures and used for the inhibition-zone and broth-culture antibacterial tests. The beef extract peptone agar medium was sterilized at 121 °C (autoclaved) before being poured into sterile Petri dishes. After sterilization, the culture medium was poured into sterile Petri dishes, followed by cooling and curing. A 200 μL bacterial suspension was aseptically transferred onto the agar surface and spread evenly. The sponge specimens were sterilized (UV irradiation for 15 min per side) before testing and then placed on the inoculated agar plates. Finally, the dishes were covered and placed in a static bacterial incubator at 37 °C for 24 h to observe bacterial growth. Because the inhibition-zone assay is diffusion-governed, it primarily reflects the ability of antibacterial species to migrate through the agar matrix and is therefore most appropriate as a qualitative screening method for solid samples tested under identical conditions. To avoid over-interpreting inhibition-zone diameters when diffusivity differs among materials, we complemented this test with a broth-based antibacterial assay (OD_600_), in which the sponges were incubated with bacterial suspensions and bacterial growth was monitored over time. Accordingly, inhibition-zone results and OD_600_ data are discussed as complementary evidence for antibacterial performance in solid-contact and liquid-culture environments, respectively.

OD_600_ was monitored using a broth-culture assay as follows: 10 mL of bacterial solution and an equivalent amount of sponge were added to well plates. The plates were then cultured at 37 °C for 7 days. The remaining bacterial solution was assessed by measuring OD_600_ using an ultraviolet spectrophotometer (Shimadzu, UV2700-I, Kyoto, Japan) every 24 h.

All antibacterial assays (inhibition-zone diameter and OD_600_ measurements) were conducted in triplicate (*n* = 3) using independently prepared samples under identical conditions. Quantitative data are reported as mean ± standard deviation. Statistical analysis was performed by one-way analysis of variance (ANOVA), and differences were considered statistically significant at *p* < 0.05.

Moreover, the bacterial morphologies were analyzed by depositing bacterial suspensions on titanium plates and observing them by SEM. The bacteria were subsequently treated with 200 μL of 2.5% glutaraldehyde. A dehydration process was carried out using ethanol concentrations of 10%, 30%, 50%, 70%, 90%, and 100% for intervals of 20 min each.

### 2.7. Cumulative Release of AgNPs

The cumulative release of AgNPs from QCH/TCNF@AgNPs was assessed using ICP-OES following previously reported methods [42,43]. A total of 100 mL of buffer solution was added to a conical bottle containing QCH/TCNF@AgNPs samples of the same mass. At 24 h intervals, 1 mL of solution was extracted from the conical flask, and 1 mL of fresh buffer solution was added to maintain a consistent total volume. The amount of AgNPs released was quantified based on the daily extracted solution. This phosphate-buffered ICP-OES release test provides a controlled baseline for comparing samples; however, real fruit-packaging environments may alter silver speciation and transport, and direct quantification of silver migration/residues in fruit tissue was beyond the scope of this study and will be evaluated in future work using food-simulant media and real-food matrices.

### 2.8. Analysis of Preservation Properties for Strawberries

Strawberries were employed as the model fruit to assess preservation properties. Strawberries of similar maturity and without visible defects were obtained from a local greenhouse farm, and within 2 h of purchase, were used to initiate the preservation test. “Day 0” was defined as the packaging day (start of storage) immediately after initial measurements; no additional chemical preservatives or post-harvest coatings were applied prior to storage, except for gentle rinsing with water and air-drying. The strawberries were washed and dried using ultrapure water before packaging. Subsequently, the samples were categorized into three groups: the control group, the group packed with PU, and the group packed with QCH/TCNF@AgNPs. For the preservation test, strawberries were placed in identical empty plastic boxes, and cushioning pads were positioned both beneath and above the fruits. As the commercial control, polyurethane (PU) pads (used as a cushioning packaging material) were used, whereas for the experimental group, the PU pads were replaced by the as-prepared composite sponge pads; the boxes were then stored at room temperature and ambient humidity. Throughout the packaging process, changes in strawberry properties were closely monitored. The weight loss of strawberries was calculated by Equation (7):

(7) $$\mathrm{Weight}\;\mathrm{loss}\;\left(\%\right)=\frac{{\mathrm{ML}}_{0}-{\mathrm{ML}}_{t}}{{\mathrm{ML}}_{0}}\times 100$$

ML_0_ denotes the initial mass of strawberries, while ML_t_ represents the mass subsequent to packaging and storage. These values were replicated three times within identical conditions.

Strawberry firmness was evaluated using a texture analyzer (Stable Micro Systems, Godalming, UK). The procedure involved positioning the sample onto the testing platform and subjecting it to compression using a P2 probe, which had a 1 mm diameter. The operational parameters of the probe were set as follows: an initial speed of 60 mm/min, a cutting speed of 8 mm/min, and a withdrawal speed of 120 mm/min. This process was performed in triplicate under consistent conditions.

The rotting rate was assessed using a modified approach. The number of decayed fruit due to fungal or microbial infections was recorded at consistent time intervals and quantified as a percentage. If the affected area was below 50% of a sample, it was assigned a value of 0.5, whereas if it exceeded 50%, it was assigned a value of 1. To minimize errors, ten samples from each group were subjected to replication within identical conditions.

The pH of strawberries was determined by a pH meter (Mettler Toledo, Greifensee, Switzerland). The relative electrical conductivity of strawberries was determined by an electrical conductivity meter (Shanghai Lei Magnetic Instrument Co., Ltd., PHS-3G, Shanghai, China). Relative electrical conductivity (electrolyte leakage) was determined by mixing 4 g of homogenized pulp with 50 mL of deionized water. The conductivity was recorded as C_0_ after stirring; then, the mixture was shaken at 100 rpm for 1 h and recorded as C_60_, followed by heating at 121 °C for 20 min and cooling to 25 °C to obtain C_121_. Relative electrical conductivity (%) was calculated using Equation (8):

(8) $$\mathrm{Relative}\;\mathrm{electrical}\;\mathrm{conductivity}\;\left(\%\right)=\frac{C_{60}-C_{0}}{C_{121}}\times 100$$

Vitamin C content was determined by the 2, 6-dichlorophenolindolephenol (DCPIP) titration method. Strawberry pulp was extracted with an oxalic acid solution, and the filtrate was titrated with DCPIP to a stable light pink endpoint. Vitamin C content was calculated according to Equation (9) and expressed as mg per 100 g fresh weight:

(9) $$X=\frac{(V-V_{0})\;\times \;T\times A}{m}\times 100$$

X is the content of L(+)-ascorbic acid in the sample (mg per 100 g); V is the volume (mL) of 2,6-dichlorophenolindophenol solution consumed in titrating the sample; V_0_ is the volume (mL) consumed in the blank titration; T is the titer of the 2,6-dichlorophenolindophenol solution, expressed as mg of ascorbic acid equivalent per mL of solution (mg/mL); A is the dilution factor; and *m* is the sample mass (g).

### 2.9. Cytotoxicity Evaluation

Mouse lung fibroblast cells (L929, ATCC CCL-1, provided by Shanghai Cell Bank, Chinese Academy of Sciences) were used for the experiments. The cytotoxicity of the composite sponge was assessed using Cell Counting Kit-8 (CCK-8, Shanghai Beitai Biotechnology Co., Ltd., Shanghai, China). First, all samples were subjected to comprehensive ultraviolet sterilization for 20 min. After that, the samples were soaked in serum-free cell medium for 24 h to obtain composite membrane leach solutions with concentrations of 0.1, 1, and 10 mg/mL, respectively. Cell viability and growth were evaluated 24 h after inoculation, and a three-time average was used to reduce error. Fluorescence images of live/dead cell experiments were also obtained.

## 3. Results and Discussion

Because the cushioning performance and preservation efficacy of packaging sponges are strongly governed by their microstructure and physical properties, we performed a systematic physical characterization to establish clear structure–property–function relationships. Morphological and network analyses (SEM and X-ray CT) were used to verify the formation of an interconnected porous architecture, while porosity/density and water-related metrics (swelling, moisture absorption, and solubility) were quantified to evaluate moisture management and structural stability under packaging-relevant conditions. Mechanical resilience (cyclic compression and firmness) was assessed to ensure recoverability under repeated deformation, and thermal analysis (TGA/DTG/DSC) was conducted to examine the thermal stability of the composite sponges. These physical datasets provide the basis for interpreting Ag release, antibacterial performance, and degradability in the subsequent sections.

### 3.1. Structure and Morphology Characterization of QCH/TCNF@AgNPs

**Figure 2:**
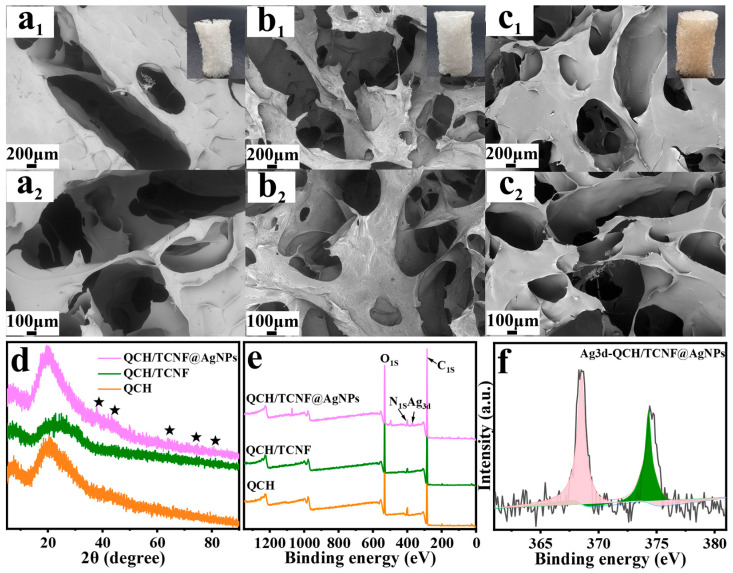
Surface morphology of QCH (**a_1_**,**a_2_**), QCH/TCNF (**b_1_**,**b_2_**), and QCH/TCNF@AgNPs (**c_1_**,**c_2_**); XRD pattern (**d**); XPS spectra (**e**); Ag_3d_ of XPS spectra (**f**); The XRD peaks marked with the symbol (★) correspond to f.c.c. Ag.

The incorporation and distribution of AgNPs in the composite sponge were verified by SEM/EDS, XRD, XPS, and X-ray CT analyses. Figure 2a_1_,c_2_ show the surface morphologies of QCH, QCH/TCNF, and QCH/TCNF@AgNPs. As shown, all three sponges exhibited honeycomb-like porous structures. On the surface, the pristine QCH sponge exhibited larger, partially closed pores than QCH/TCNF and QCH/TCNF@AgNPs. In contrast, QCH/TCNF and QCH/TCNF@AgNPs had denser structures and exhibited abundant through-hole channels, which were beneficial for softness and elasticity. The internal through-hole channels of the different sponges can also be observed in Figure S1. The diameter of channels in QCH/TCNF and QCH/TCNF@AgNPs was about 1 mm, and the depth was generally greater than that in QCH. The inset morphologies also proved that QCH/TCNF and QCH/TCNF@AgNPs had strong structures due to the addition of TCNF fibers and AgNPs. The abundant hydroxyl groups on TCNF facilitate hydrogen bonding with QCH, resulting in a denser framework with more interconnected (through-hole) pores.

Figure 2d shows the XRD spectrum of different sponges. As shown, the QCH/TCNF@AgNPs exhibited the characteristic diffraction peaks at 38.2°, 44.4°, 64.6°, 77.4°, and 81.6°, which were consistent with the standard powder XRD pattern for f.c.c. Ag (ICDD PDF No 00-004-0783). The XRD spectrum proved the successful synthesis of AgNPs. Furthermore, the XPS analysis (Figure 2e,f) also confirmed the presence of AgNPs.

**Figure 3:**
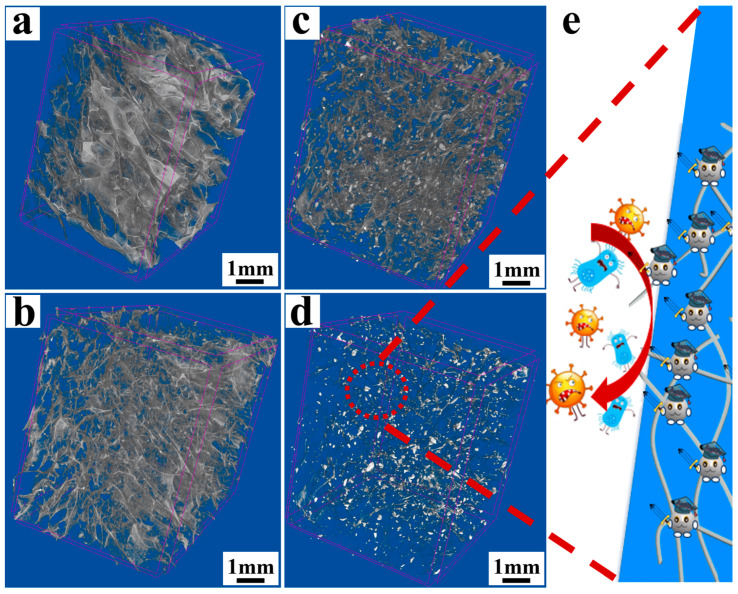
X-ray computed tomography (CT) (acquired using an X-ray CT system) images of QCH (**a**); QCH/TCNF (**b**) and QCH/TCNF@AgNPs (**c**,**d**); antibacterial diagram of AgNPs (**e**); The red dashed line delineates the antibacterial mechanism of the AgNPs in the schematic diagram.

The composite sponge was further characterized by X-ray CT, as shown in Figure 3. As shown in Figure 3a, the structure of QCH was more lamellar with large pores, and it had almost no 3D through-hole network structure. The addition of TCNF made the sponge more porous and obviously exhibited a through-hole network structure due to the intertwining between TCNF and QCH fibers, which was shown in Figure 3b. After adding TCNF@AgNPs, the composite sponge not only maintained the 3D through-hole network structure, but AgNPs were also uniformly attached to the skeleton of the composite sponge, as shown in Figure 3c. Furthermore, more metal nanoparticles could be directly obtained by changing the light source in X-ray CT, as shown in Figure 3d. As simulated in Figure 3e, once the composite sponge is used in the packaging process, the immobilized AgNPs can be released and kill bacteria, thereby improving preservation.

### 3.2. Characterization of Physical Properties

**Figure 4:**
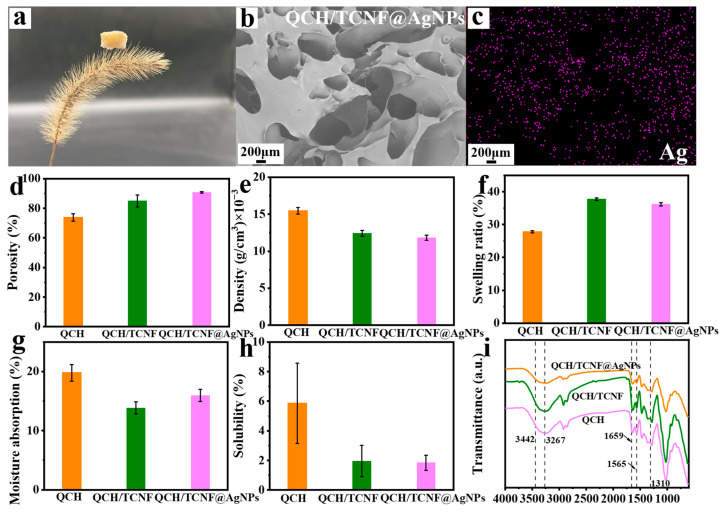
A photograph of QCH/TCNF@AgNPs (**a**); the surface and EDS graph of Ag element in QCH/TCNF@AgNPs (**b**,**c**); physical properties analysis of composite sponge: porosity (**d**); density (**e**); swelling ratio (**f**); moisture absorption (**g**); solubility (**h**); and FT-IR spectra (**i**).

The physical properties of composite sponges were further investigated, as illustrated in Figure 4. Figure 4a demonstrates the lightweight nature of QCH/TCNF@AgNPs, as the sponge can be readily supported by a foxtail grass spikelet. And it can also be supported by stamens, as shown in Figure S2. In Figure 4b,c, the EDS graphs of QCH/TCNF@AgNPs further demonstrate the presence and distribution of silver element, which were consistent with the results of the XPS spectra and X-ray CT. The EDS graphs of C, N, and O are also shown in Figure S3. Ag was uniformly distributed on the sponge skeleton without obvious aggregation, consistent with the SEM/EDS observations. Compared to QCH/TCNF and QCH/TCNF@AgNPs, the porosity of QCH (73.88%) was the lowest. The addition of TCNF and TCNF@AgNPs led to an increase in porosity to 84.94% and 90.71%, respectively (Figure 4d). The density of QCH/TCNF@AgNPs was also the lowest and reached 0.0118 g/cm^3^, as shown in Figure 4e. Compared to QCH (27.84%), the swelling rate of QCH/TCNF and QCH/TCNF@AgNPs increased to 37.75% and 36.18%, respectively (Figure 4f). The modified porous structures were beneficial for softness and elasticity. Moreover, the moisture absorption rate was relevant to the soft structures, and decreased from 19.78% (QCH) to 13.83% (QCH/TCNF) and 15.97% (QCH/TCNF@AgNPs) (Figure 4g). The absorbent property of QCH/TCNF@AgNPs is also exhibited in Video S1. The water solubility of the composite sponge was also analyzed in Figure 4h. It was found that the water solubility of QCH/TCNF and QCH/TCNF@AgNPs decreased with the addition of TCNF and TCNF@AgNPs, which was mainly attributed to the hydrogen bonding between TCNF and QCH fibers.

FT-IR spectroscopy was used to analyze potential structural changes in the composite sponge (Figure 4i). The broad absorption band at 3357 cm^−1^ in QCH was attributed to the overlap of –OH and –NH stretching vibrations arising from alcohol and amine groups [44]. The bands at 2918 cm^−1^ and 2873 cm^−1^ correspond to C–H stretching vibrations of the polysaccharide backbone. The bands in the 1650–1550 cm^−1^ region (e.g., ~1639 and 1583 cm^−1^) can be attributed to amide I/II vibrations associated with residual acetylated units in the chitin-based substrate, potentially with overlap from bound water and polysaccharide vibrations. The feature at 1374 cm^−1^ is related to C–O vibrations of the polysaccharide framework. The peaks at 1056 cm^−1^ and 1026 cm^−1^ are related to symmetric stretching of C–O–C and skeletal vibrations of the polysaccharide framework, respectively. Overall, the absence of new characteristic absorption bands suggests that the primary chemical structure of QCH is largely preserved after incorporating TCNF (or TCNF@AgNPs). Here, good compatibility refers to the favorable interfacial affinity and homogeneous integration between TCNF and QCH, which can be attributed to their similar polysaccharide backbones and abundant polar groups enabling hydrogen bonding and electrostatic interactions. Subtle spectral differences may be masked by band overlap and the relatively low loading of the nanocellulose component [45].

**Figure 5:**
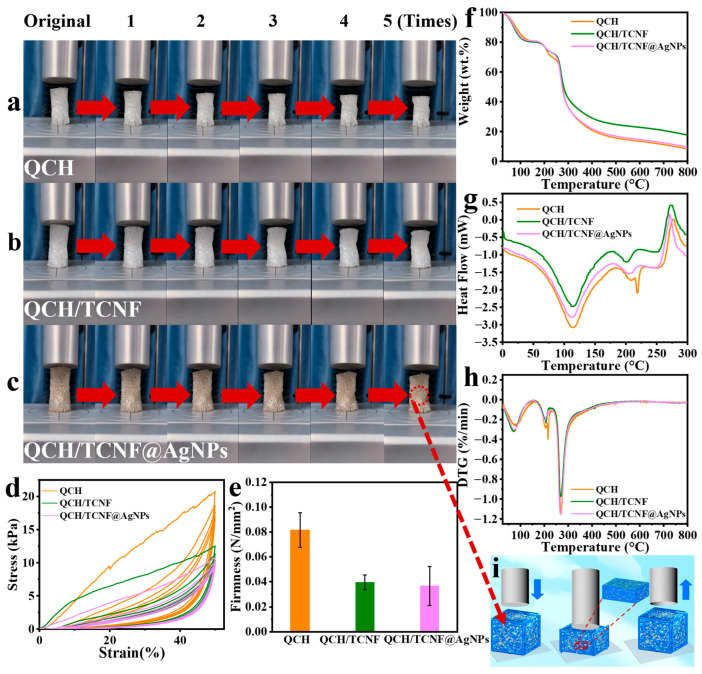
Cyclic compression process (**a**–**c**), curves (**d**), firmness (**e**), thermograms pattern (**f**), DSC pattern (**g**), DTG pattern (**h**), of QCH, QCH/TCNF, and QCH/TCNF@AgNPs; schematic diagram of cyclic compression (**i**); The red dashed line delineates the compression and recovery process of the sample in the schematic diagram.

The mechanical properties of the composite sponge were analyzed using cyclic compression curves, as shown in Figure 5. Figure 5a–c illustrate the changes of the three different sponges during the entire compression cycle. After five compression cycles, three composite sponges returned to their original shape and size, which demonstrated their softness and elasticity (Figure 5i). As shown in Figure 5d, the compression cycle curve of QCH/TCNF@AgNPs was similar to that of QCH/TCNF. Under the same force, QCH/TCNF and QCH/TCNF@AgNPs could recover their original shapes, while QCH needed more force. The compressive tests ensured the soft and elastic structural characteristics of QCH/TCNF@AgNPs, which were consistent with the results of porosity and swelling ratio. With the addition of TCNF and TCNF@AgNPs, the through-hole structures of the composite sponge became more stable due to hydrogen bonding. Compared to QCH in Figure 5e, the firmness of QCH/TCNF@AgNPs decreased by 55.03%. The firmness results also supported the analysis of cyclic compression as shown in Figure 5d. The softness of QCH/TCNF@AgNPs could also be observed in Video S2. The above data indicated that the composite sponge was suitable for practical application as a fruit packaging material due to its softness and elasticity.

TGA and derivative thermogravimetry (DTG) curves of three different sponges are displayed in Figure 5f,g. As observed in Figure 5f, the weight loss rate of QCH was approximately 91.57% at 75 °C in the initial stage, whereas that of QCH/TCNF and QCH/TCNF@AgNPs was approximately 88.49% and 89.50%, respectively. This was attributable to hydrogen binding between the two fibers and the higher water retention of TCNF. A sudden increase in the weight loss rate of QCH was noted within the temperature range of 200 °C to 250 °C, while the weight loss of QCH/TCNF and QCH/TCNF@AgNPs was less pronounced. This change was a result of the incorporation of TCNF and TCNF@AgNPs, which proportionally reduced the QCH content and, thus, improved the thermal stability of the composite sponge. This transformation was also evident in the DSC curve (Figure 5h).

### 3.3. Degradable Performance Analysis

**Figure 6:**
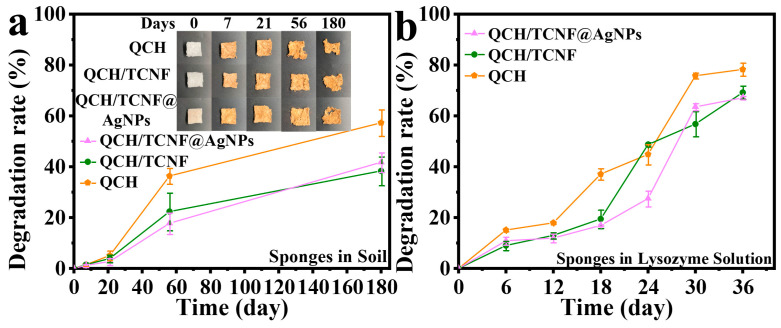
Degradation ratio (mass loss) of the composite sponge in natural soil (**a**) and lysozyme solution (**b**).

Conventional petroleum-based packaging materials are generally non-biodegradable and raise environmental concerns [46,47]. Figure 6 shows the degradation of composite sponges in natural soil and lysozyme solution. It can be seen from Figure 6a that all composite sponges gradually degraded in natural soil due to the biodegradable raw materials. The degradation trends of QCH, QCH/TCNF, and QCH/TCNF@AgNPs were similar, and the degradation rates were 57.26%, 38.33%, and 41.75% after 180 days, respectively. Representative photographs of the sponges during soil burial are also shown in Figure 6a. Therefore, once the composite sponge is used up, it can degrade in natural soil. As shown, the degradation rate of QCH/TCNF@AgNPs had a slower increasing trend than that of QCH/TCNF because the immobilized AgNPs released slowly and modified the denser structure of the composite sponge. The accumulation of AgNPs in natural soil is also an important concern; in fact, the released AgNPs were transformed into silver sulfide in the presence of the chemical composition of natural soil [48,49].

Furthermore, the degradation rate of the composite sponge in lysozyme solution was also analyzed in Figure 6b. As exhibited, the overall degradation rate of the three sponges was faster than that in natural soil, and all samples exceeded 50% after 36 days. The degradation rate of QCH/TCNF and QCH/TCNF@AgNPs after 36 days was 69.19% and 67.09%, respectively. The degradation rate of QCH was faster than that of QCH/TCNF and QCH/TCNF@AgNPs because the composition of QCH was simpler and the structure was also less compact. In general, the degradation rate of the three composite sponges in natural soil was lower than that in the enzyme solution. This may be because the enzyme solution more easily penetrated the interior and rapidly contacted the structure of the composite sponge, which was conducive to degradation.

### 3.4. Silver Release and Antibacterial Performance

**Figure 7:**
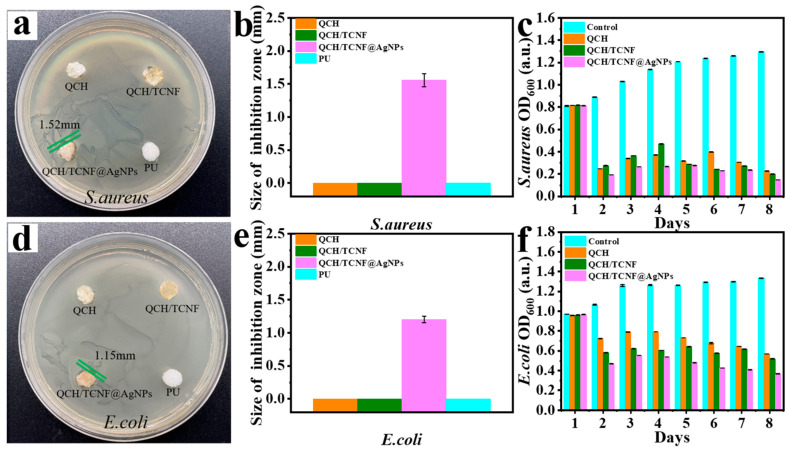
The antibacterial activity against *S. aureus*: (**a**,**b**) pictures and size of the inhibition zone; (**c**) OD_600_ values. The antibacterial activity against *E. coli*: (**d**,**e**) pictures and size of the inhibition zone; (**f**) OD_600_ values.

The main microorganisms selected for the experiment were the representative Gram-negative bacterium *E. coli* and the Gram-positive bacterium *S. aureus*. Figure 7 illustrates the antibacterial activity of the composite sponge.

It should be noted that inhibition-zone diameters are diffusion-dependent and are therefore interpreted here as qualitative support, while OD_600_ measurements provide antibacterial evidence under liquid-culture conditions. As is well known, AgNPs primarily exert antibacterial effects through physical methods that kill bacteria, and the antibacterial images can be seen in Figure 7a,d. As shown, the antibacterial effect of QCH and QCH/TCNF was limited because solid QCH has little antibacterial activity. But the antibacterial activity of QCH and QCH/TCNF could be obtained in a liquid state, as shown in OD_600_ values. As shown in Figure 7b,e, the antibacterial zone diameter of QCH/TCNF@AgNPs was 1.52 mm against *S. aureus* and 1.15 cm against *E. coli*. The results showed that the antibacterial activity of QCH/TCNF@AgNPs against *S. aureus* was stronger than that against *E. coli*. This difference can be attributed to the distinct cell-envelope structures of the two bacteria. Gram-negative *E. coli* possesses an additional outer membrane that can hinder the interaction/penetration of silver species, whereas Gram-positive *S. aureus* lacks an outer membrane, which may facilitate antibacterial action under the same exposure conditions.

OD_600_ values show the antibacterial activity of the composite sponge in the liquid state, as depicted in Figure 7c,f. The OD_600_ values of the control group increased over time for both bacteria, indicating continuous bacterial growth. In contrast, the QCH-, QCH/TCNF-, and QCH/TCNF@AgNPs-treated groups showed suppressed growth, evidenced by lower OD600 values. Notably, QCH/TCNF@AgNPs exhibited the strongest inhibition, consistent with the synergistic effect of contact-active QCH and immobilized Ag species. Under the single antibacterial activity of QCH, the antibacterial trends of QCH and QCH/TCNF decreased first and then increased, which could be seen in OD_600_ values in Figure 7c,f. Although the total Ag content in the QCH/TCNF@AgNPs sponge was only 0.18 wt%, the immobilized and highly dispersed AgNPs on TCNF provide extensive interfacial exposure and a controlled release effect, leading to strong antibacterial efficacy when combined with the contact-active QCH. These results demonstrate that QCH /TCNF@AgNPs exhibited extended antibacterial activity due to the synergistic antibacterial effect of AgNPs and QCH, indicating potential for antibacterial packaging applications.

### 3.5. Antibacterial Mechanism Analysis

**Figure 8:**
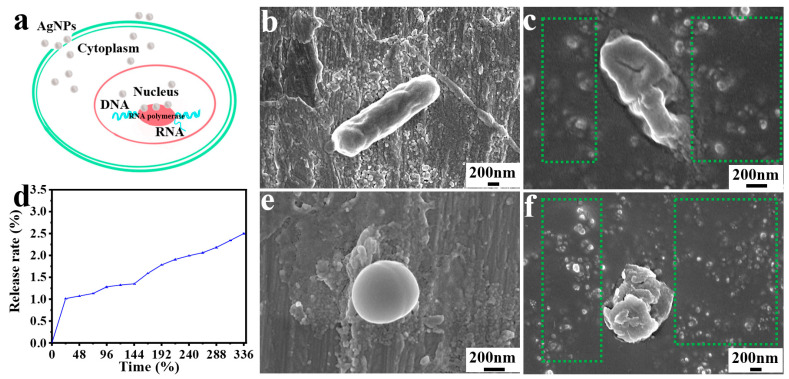
An antibacterial schematic diagram of AgNPs (**a**); bacterial surface morphologies of *E. coli* after treatment by QCH/TCNF@AgNPs (**b**,**c**); the cumulative release trend of AgNPs from QCH/TCNF@AgNPs (**d**); bacterial surface morphologies of *S. aureus* before and after treatment by QCH/TCNF@AgNPs (**e**,**f**).

Figure 8 summarizes the proposed antibacterial mechanism and the cumulative release profile of silver species from the composite sponge. Figure 8a provides a schematic illustration of how immobilized AgNPs (and released Ag species) can disrupt bacterial membranes and interfere with intracellular components, ultimately leading to cell damage and death [50,51].

To support the proposed mechanism, SEM images of bacteria before and after exposure to QCH/TCNF@AgNPs are shown in Figure 8b,c,e,f. As displayed, integrated *S. aureus* appeared as spheres with diameters of approximately 500–900 nm, while *E. coli* exhibited a rod-shaped structure with diameters around 700 nm and lengths of 2–4 μm. The surfaces of the original *E. coli* and *S. aureus* were smooth, and the bacterial morphology was intact. After being acted on by QCH/TCNF@AgNPs, the bacterial morphology was rough, shrunken, or even cracked. Moreover, the presence of AgNPs around dead bacteria was distinctly observed (delineated by dotted green lines), which proves the release and killing activity of AgNPs [52]. Future work will vary Ag loading to establish a quantitative dose–response relationship and determine the minimum effective Ag content for preservation performance while further improving cost-efficiency and safety.

During fruit preservation, the composite sponge is exposed to a moist and chemically dynamic microenvironment created by fruit respiration and exudates. Therefore, the release and stability of silver species are key considerations. The total Ag content of the QCH/TCNF@AgNPs sponge, determined by ICP-OES, was 0.18 wt% (on a dry-mass basis). Figure 8d shows the cumulative release profile of AgNP-derived silver from QCH/TCNF@AgNPs measured in phosphate buffer as a standardized medium. An initial release was observed within 0–24 h (1.01%), which can be attributed to loosely bound/near-surface silver species, followed by a much slower release over 0–336 h. After 14 days, the total cumulative release was only 2.49%, indicating controlled release enabled by immobilizing AgNPs on TCNF and embedding them within the crosslinked QCH network. In practical fruit-packaging scenarios, moisture level, acidity (pH), and fruit composition may further influence silver dissolution/complexation and nanoparticle stability [53]. Nevertheless, the low release observed in the buffer supports the robustness of the immobilization strategy and is consistent with durable antibacterial preservation performance. The design goal of this work is to immobilize AgNPs on TCNF and embed them within a crosslinked QCH network to mitigate uncontrolled leaching while retaining antibacterial efficacy. Future work will quantitatively compare immobilized versus free AgNPs under identical silver loading to further delineate diffusion- versus contact-mediated antibacterial contributions.

### 3.6. Analysis of Fresh-Keeping Properties of Strawberries

**Figure 9:**
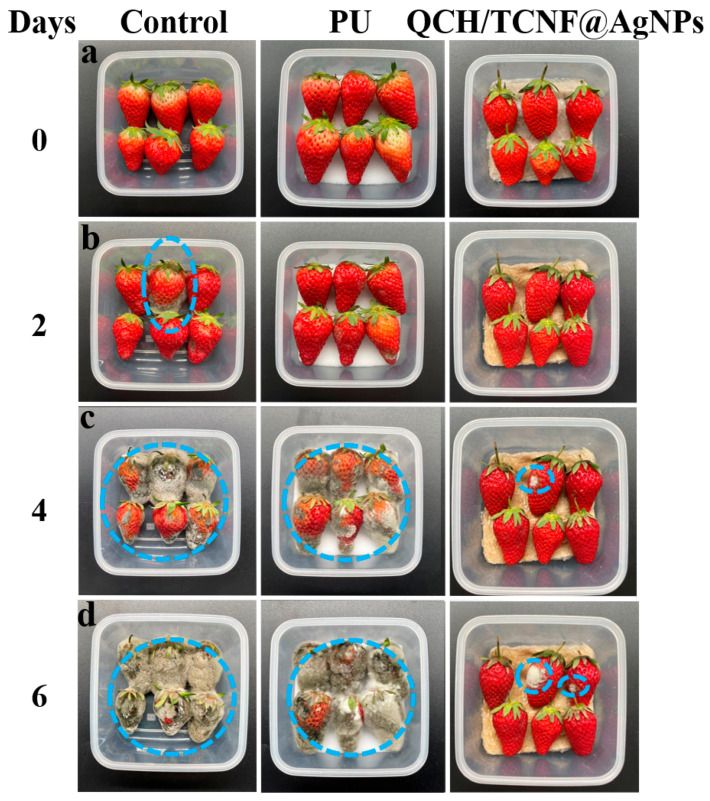
The morphological changes of strawberries after packaging with different materials. The changes in the Control group, the PU group, and the QCH/TCNF@AgNPs group at day 0 (**a**), day 2 (**b**), day 4 (**c**), and day 6 (**d**) are demonstrated. Day 0 denotes the start of storage (packaging day) immediately after initial measurements; The blue dashed line demarcates the area showing rotting progression.

Strawberries have a delicate skin that is easily damaged during transportation and storage, resulting in a short shelf life. Figure 9 shows the morphological changes of strawberries packaged with different materials during storage. Before storage, cushioning pads were placed both beneath and above the strawberries, as shown in Figure S4. For photography, the top pad was removed to provide an unobstructed view, as shown in Figure 9. After 2 days, visible mold appeared in the control and PU groups. In contrast, strawberries packaged with QCH/TCNF@AgNPs remained largely intact, which can be attributed to the combined cushioning and antibacterial protection. After 4 days, severe spoilage was observed in the control and PU groups. Meanwhile, the QCH/TCNF@AgNPs group showed only limited localized mold. In contrast, only a few strawberries in the QCH/TCNF@AgNPs group showed minor mold, and the overall appearance (color and shape) was better maintained.

**Figure 10:**
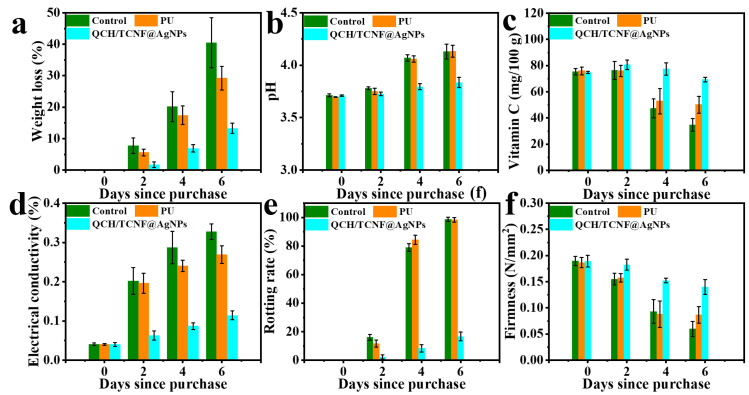
The chemical parameter changes of strawberries packaged with different materials. Weight loss (**a**); pH (**b**); vitamin C (**c**); electrical conductivity (**d**); rotting rate (**e**); and firmness (**f**).

Figure 10 also displays changes in the chemical parameters of strawberries during storage, including weight loss, electrical conductivity, vitamin C, pH, rotting rate, and firmness. As shown in Figure 10a, the weight loss of strawberries increased over time. On day 6, the weight loss of the control group was approximately three times higher than that of the QCH/TCNF@AgNPs group, likely due to the weaker moisture-buffering and protection in the absence of the composite sponge. In addition, the pH of the QCH/TCNF@AgNPs group increased more slowly than that of the control and PU groups, indicating better preservation of chemical quality during storage (Figure 10b). During storage, the consumption of organic acids and nutrients can contribute to an overall increase in pH [54]. As shown in Figure 10c, the vitamin C content in the QCH/TCNF@AgNPs group remained at a relatively high level over the storage period. Relative electrical conductivity (Figure 10d), which reflects membrane integrity, increased more slowly in the QCH/TCNF@AgNPs group, suggesting better cellular integrity. As shown in Figure 10e, only 16.66% of strawberries in the QCH/TCNF@AgNPs group exhibited decay after 6 days, whereas the control and PU groups were nearly completely spoiled (~100%). Consistently, the firmness of strawberries packaged with QCH/TCNF@AgNPs was better maintained over time (Figure 10f), while the control and PU groups showed a much more pronounced decline, in agreement with the observed decay and electrolyte leakage. Overall, the photographic observations and physicochemical parameters collectively indicate that QCH/TCNF@AgNPs can effectively preserve strawberries for up to 6 days under the tested conditions.

### 3.7. Cytocompatibility Analysis

**Figure 11:**
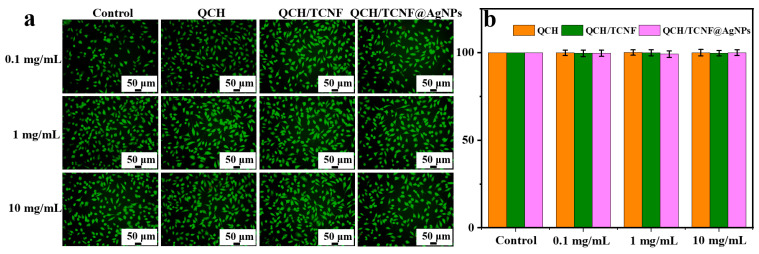
Fluorescence images of cells, live/ dead assay (**a**); cell viability treated by different composite sponges (**b**).

Figure 11 presents the cytocompatibility results for QCH, QCH/TCNF, and QCH/TCNF@AgNPs. A packaging sponge intended for food-contact applications should exhibit minimal cytotoxicity [55,56]. As shown, all extract groups displayed high cell viability. Even at an extract concentration of 10 mg/mL, cell viability remained high, consistent with normal cell morphology and the Live/Dead observations. The 10 mg/mL extract was selected for the Live/Dead assay, where most cells showed healthy morphology with clear boundaries (Figure 11a). In addition, the viability values for QCH, QCH/TCNF, and QCH/TCNF@AgNPs were comparable to the control group (Figure 11b), indicating no appreciable cytotoxicity under the tested extract conditions. Overall, these results support the good cytocompatibility of the composite sponge and suggest its suitability for fruit-packaging applications.

## 4. Conclusions

In summary, this work demonstrates a composite sponge (QCH/TCNF@AgNPs) that integrates cushioning performance with durable synergistic antibacterial activity. The sponge was fabricated by incorporating TEMPO-oxidized cellulose nanofibers-immobilized Ag species into a quaternized chitin matrix, yielding a soft and elastic porous structure suitable for packaging. The composite exhibited effective antibacterial activity against both *E. coli* and *S. aureus*, while maintaining controlled silver release (2.49% after 14 days in phosphate buffer) and good biodegradability (mass loss exceeding 50% after 36 days in lysozyme solution). In addition, cytocompatibility assays indicated high cell viability under the tested extract conditions. Importantly, the composite sponge improved the preservation performance of fresh strawberries and extended the effective storage period to 6 days under the evaluated conditions. Overall, this study provides a practical strategy to combine biomass-derived polysaccharides and immobilized silver species to achieve cushioning, antibacterial protection, and degradability in a single packaging sponge, highlighting its potential for fruit-preservation packaging.

## Data Availability

The original contributions presented in this study are included in the article/Supplementary Material. Further inquiries can be directed to the corresponding author.

## Supplementary Materials

The following supporting information can be downloaded at https://www.mdpi.com/article/10.3390/polym18030327/s1, Figure S1: The internal SEM image of composite sponge; Figure S2: The stamens can bear a certain weight of the composite sponge; Figure S3: The EDS graph for QCH/TCNF@AgNPs (include C; N; O; Ag); Figure S4: Image of sponge covering during strawberry storage; Video S1: The absorbent property of QCH/TCNF@AgNPs; Video S2: The softness of QCH/TCNF@AgNPs.
