# Supplementary material for: TEMPO-Oxidized Nanocellulose In Situ-Immobilized AgNPs-Modified Chitin-Based Composite Sponge for Synergistic Antibacterial Fruit Preservation
Source: Polymers (Basel). 2026 Jan 26;18(3):327. doi: 10.3390/polym18030327 (PMC12899400; doi:10.3390/polym18030327)
Supplement: Supplementary file 1 [file polymers-18-00327-s001.zip › Supporting information.pdf]

Supporting Information

TEMPO-Oxidized Nanocellulose In Situ-Immobilized AgNPs-Modified Chitin-Based Composite Sponge for Synergistic Antibacterial Fruit Preservation

Zijun Zhang, Qi Zhang, Qimeng Jiang * and Hao Ma

State Key Laboratory of Green Papermaking and Resource Recycling, Qilu University of Technology, Jinan 250353, China

* Correspondence: qmj@qlu.edu.cn

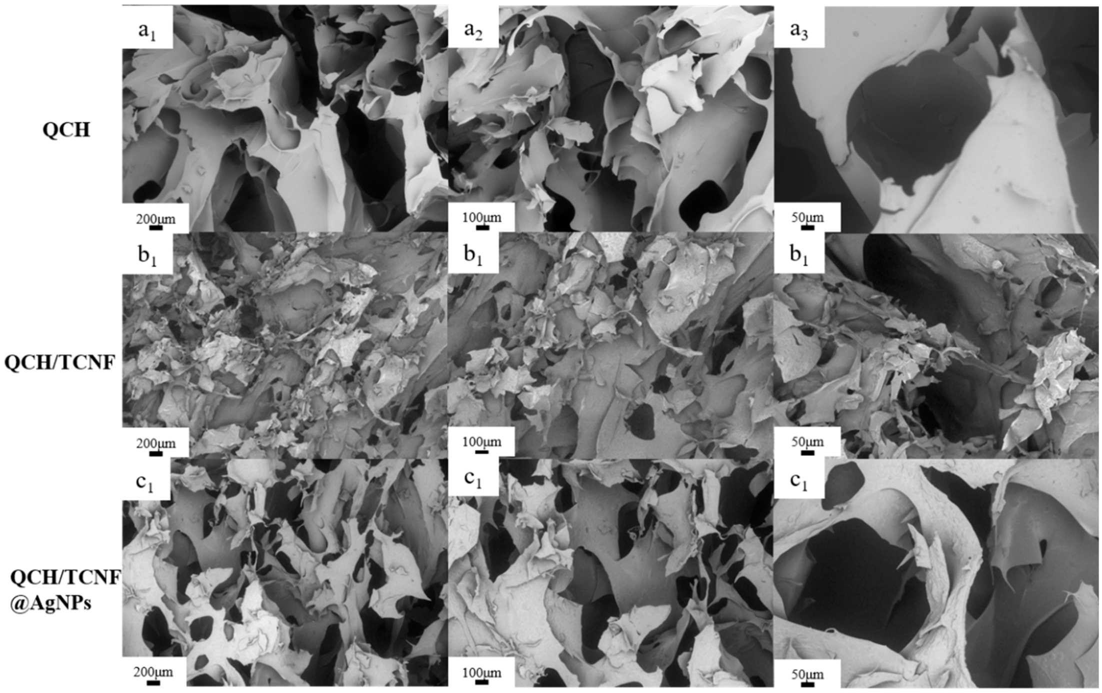

Figure S1. The internal SEM image of composite sponge.

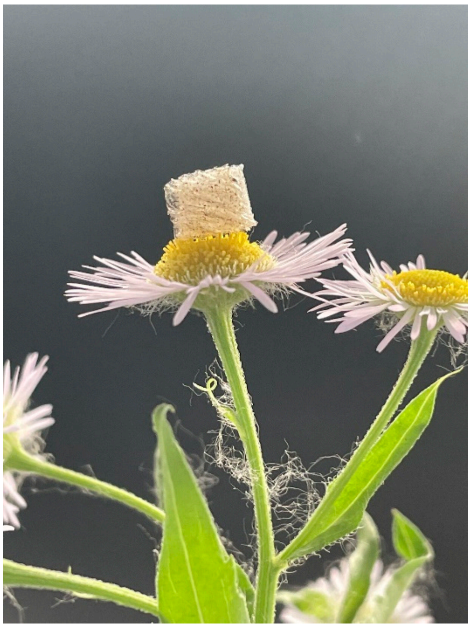

Figure S2. The stamens can bear a certain weight of the composite sponge.

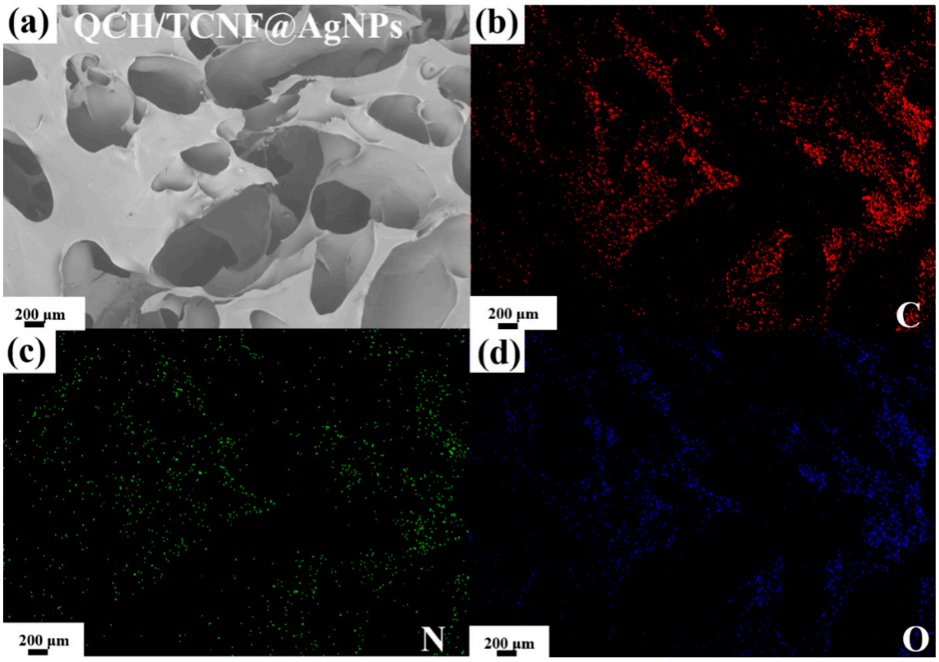

Figure S3. The EDS graph for QCH/TCNF@AgNPs (include C; N; O; Ag)

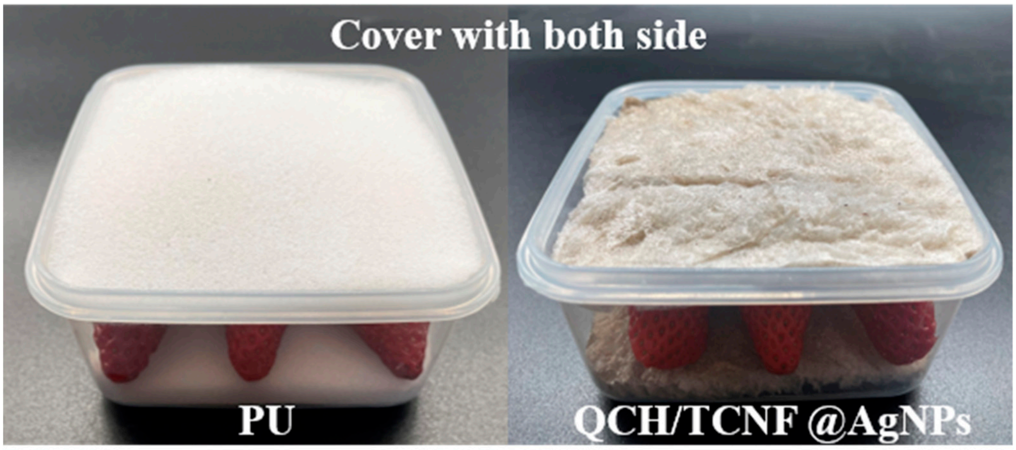

Figure S4. Image of sponge covering during strawberry storage
